# Editorial: Role of endophytic/symbiotic fungi in plant growth promotion and disease suppression

**DOI:** 10.3389/ffunb.2026.1820241

**Published:** 2026-03-17

**Authors:** Gustavo Santoyo, Ajay Kumar, Ma. del Carmen Orozco-Mosqueda, Gilberto de Oliveira Mendes

**Affiliations:** 1 Institute of Chemical and Biological Research, Universidad Michoacana de San Nicolás de Hidalgo, Morelia, Mexico; 2 Amity Institute of Biotechnology, Amity University, Noida, India; 3 Department of Biochemical and Environmental Engineering, National Technological Institute of Mexico in Celaya, Celaya, Mexico; 4 Instituto de Ciências Agrárias, Universidade Federal de Uberlândia, Monte Carmelo, Brazil

**Keywords:** biocontrol, fungal diversity, plant growth-promoting fungi, plant microbiome, sustainable agriculture

## Introduction

Plants can associate with endophytic and symbiotic fungi to enhance their fitness and survive under diverse environmental conditions, including abiotic stresses such as drought, salinity, extreme temperatures, nutrient deficiency, and soil contamination, as well as biotic stresses caused by pathogens and herbivores (Cortez-Lázaro et al., 2026; Peng et al., 2026; Taheri, 2025). These interactions improve nutrient acquisition, modulate hormonal responses, induce systemic resistance, and strengthen antioxidant mechanisms, thereby contributing to greater plant adaptation and productivity across different agricultural systems (Santillan-Culquimboz et al., 2025). Various bioinoculants are based on endophytic or symbiotic fungi as plant growth-promoting agents, biocontrol agents against pests and pathogens, and participants in processes that contribute to the environmental cleaning of toxic metals and metalloids (Yousuf, 2026; Philip et al., 2025; Santillan-Culquimboz et al., 2025).

Endophytic fungi establish mutualistic or commensal interactions with their plant hosts and are defined by their ability to colonize and persist within internal plant tissues (such as roots, stems, leaves, or the phyllosphere) without causing apparent damage (Waqar et al., 2024). They may also be vertically transmitted through seeds to subsequent generations. Symbiotic fungi, in contrast, form close associations with living plant hosts by colonizing roots, stems, or leaves, resulting in reciprocal benefits (Gomes et al., 2018; Chen et al., 2025). In the case of arbuscular mycorrhizal fungi (AMF), evidence suggests that these microorganisms have cooperated with plants for approximately 400 million years (Van Der Heijden, 2010). They are mainly associated with trees and shrubs and colonize root tissues, enhancing nutrient and water uptake and thereby improving host performance (Orozco-Mosqueda et al., 2025).

In one of the studies published on this topic, Zhang et al. analyzed the interactions between the arbuscular mycorrhizal fungus *Rhizophagus irregularis* and *Trichoderma harzianum*, inoculated individually or in combination, to suppress Fusarium wilt in tomato plants. Both fungi significantly reduced pathogen colonization in roots, although co-inoculation did not produce an additional suppressive effect. Pathogen infection activated jasmonic acid (JA)-dominated defenses, whereas beneficial microorganisms reprogrammed the immune response toward a salicylic acid (SA)-associated pathway, characterized by increased SA accumulation and enhanced phenylalanine ammonia-lyase (PAL) and polyphenol oxidase (PPO) activities. These changes were negatively correlated with pathogen abundance. The results indicated that disease suppression was based on a hormonal shift from JA- to SA-mediated defenses, without additive benefits from fungal combination. This study provides evidence of hormonal trade-offs underlying fungus-induced resistance and offers a basis for the rational design of microbial consortia for sustainable disease management.

In another study, Liu et al. evaluated the role of AMF in rhizospheric interactions within an intercropping system of *Nicotiana tabacum* and maize (*Zea mays*). Through pot experiments, 16S rDNA and ITS sequencing, and widely targeted metabolomics using Liquid Chromatography–Tandem Mass Spectrometry (LC-MS/MS), the authors assessed changes in soil microbiota, rhizosphere metabolites, and agrochemical properties under mycorrhizal colonization. AMF inoculation promoted plant growth and improved nutrient uptake, significantly increasing alkaline-hydrolyzable nitrogen, available phosphorus, and potassium in the tobacco rhizosphere. Intercropping enhanced the relative abundance of genera such as *Penicillium*, *Trichoderma*, *Blastomonas*, and *Sphingomonas*, which were associated with improved plant performance. Mycorrhizal inoculation and intercropping also reshaped the rhizosphere metabolite profile, while pH and organic matter were identified as key drivers of microbial community structure. Overall, AMF-mediated rhizospheric interactions optimized nutrient availability and regulated plant growth in associated cropping systems.

Lu et al. demonstrated for the first time that *Massilia consociata* KC 009 (a Gram-negative bacterium belonging to the family Oxalobacteraceae) significantly promotes rooting in chrysanthemum cuttings, increasing rooting rate, root dry weight, and root number and length. Inoculation enhanced physiological indicators such as soluble proteins, soluble sugars, chlorophyll, and indole-3-acetic acid (IAA), with soluble protein being the factor most closely associated with root formation. Importantly, KC 009 reshaped the endophytic microbiome, reducing overall diversity and shifting bacterial dominance toward *Chryseobacterium* and *Alcaligenes*, genera linked to IAA production and stress tolerance. Notably, the abundance of *Cladosporium*, a potentially pathogenic endophytic fungus, significantly decreased, suggesting an indirect biocontrol effect. Correlation and functional prediction analyses indicated that changes in both bacterial and fungal endophytic communities were closely related to metabolites associated with plant growth. These findings highlight that rooting promotion depends not only on the direct bacterial effect but also on endophytic microbiome reorganization, including the suppression of potentially harmful fungi.

Entomopathogenic fungi such as *Beauveria bassiana* not only act as biological control agents against insects but can also establish endophytic associations and communicate with plants through volatile organic compounds (VOCs) (Crespo et al., 2008). In the study by Adame-Garnica et al., VOCs emitted by two strains of *B. bassiana* (AS5 and AI2), as well as 3-methylbutanol (3MB), the most abundant fungal volatile, were evaluated in sorghum plants. Exposure to these volatiles stimulated plant growth, promoted reactive oxygen species (ROS) and IAA accumulation, and increased levels of SA, JA, and phenolic compounds. Additionally, enhanced expression of the defense-related genes *SbPR-1* and *SbCOI1* was observed. VOCs from strain AS5 induced stronger biochemical responses than those from AI2, indicating strain-specific effects. These findings demonstrate that *B. bassiana* volatiles, particularly 3MB, can simultaneously promote growth and activate adaptive defense mechanisms, underscoring the role of fungal VOCs as key modulators of plant physiology and immunity.

## Conclusion

Collectively, these studies demonstrate that endophytic and symbiotic microorganisms, through direct colonization, microbiome restructuring, nutrient mobilization, hormonal reprogramming, and volatile-mediated signaling, play a central role in enhancing plant growth and resilience. Understanding the mechanistic basis of these interactions provides critical insights for the development of next-generation bioinoculants and sustainable agricultural strategies aimed at improving crop productivity and stress tolerance under a changing climate.

## References

[B1] Chen D. Yang J. Wang S. Lan S. Wang Y. Liu Z. J. . (2025). Comparative analysis of community composition and network structure between phyllosphere endophytic and epiphytic fungal communities of Mussaenda pubescens. Microbiol. Spectr. 13, e01019–e01024. doi: 10.1128/spectrum.01019-24, PMID: 39625383 PMC11705847

[B2] Cortez-Lázaro A. A. Chavez-Castillo J. I. Romero-Bozzetta J. L. Rojas-Paz J. L. Manes-Cangana G. A. Cortez-Lázaro R. A. . (2026). From mechanisms to networks: A global bibliometric and functional synthesis of Trichoderma in plant disease management. J. Agric. Food Res., 102706. doi: 10.1016/j.jafr.2026.102706, PMID: 41815951

[B3] Crespo R. Pedrini N. Juárez M. P. Dal Bello G. M. (2008). Volatile organic compounds released by the entomopathogenic fungus *Beauveria bassiana*. Microbiological Res. 163, 148–151. doi: 10.1016/j.micres.2006.03.013, PMID: 16733086

[B4] Gomes T. Pereira J. A. Benhadi J. Lino-Neto T. Baptista P. (2018). Endophytic and epiphytic phyllosphere fungal communities are shaped by different environmental factors in a Mediterranean ecosystem. Microbial Ecol. 76, 668–679. doi: 10.1007/s00248-018-1161-9, PMID: 29500493

[B5] Orozco-Mosqueda M. D. C. Glick B. R. Santoyo G. (2025). Cross-talk within plant niches: endophytic and arbuscular mycorrhizal fungi for sustainable crop production. FEMS Microbiol. Rev., fuaf063. doi: 10.1093/femsre/fuaf063, PMID: 41388904 PMC12766460

[B6] Peng L. Yang Y. Martin F. M. Yuan Z. (2026). Endophytes with mycorrhizal potentials: biological and ecological implications. New Phytol. 249, 2224–2231. doi: 10.1111/nph.70772, PMID: 41261810

[B7] Philip B. Eid A. R. Bayoumi S. R. Heflish A. Atallah O. O. Abdelwahab E. A. . (2025). Synergistic curative effects of *Trichoderma hamatum* and *Rumex dentatus* against Alternaria alternata, the causal agent of tomato leaf spot disease. Front. Plant Sci. 16. doi: 10.3389/fpls.2025.1700051, PMID: 41451272 PMC12728584

[B8] Santillan-Culquimboz H. W. Leiva S. T. Munoz-Salas M. N. Meza-Maicelo W. Lozano-Isla F. Oliva-Cruz M. . (2025). Ecology and functions of *Trichoderma* in coffee and cocoa agroecosystems: bibliometric and systematic insights for sustainable agriculture. Front. Microbiol. 16. doi: 10.3389/fmicb.2025.1717484, PMID: 41395480 PMC12698567

[B9] Taheri P. (2025). Endophytic fungi as regulators of phytohormones production: Cytomolecular effects on plant growth, stress protection and importance in sustainable agriculture. Plant Stress, 100978. doi: 10.1016/j.stress.2025.100978, PMID: 41815951

[B10] Van Der Heijden M. G. A. (2010). Mycorrhizal fungi reduce nutrient loss from model grassland ecosystems. Ecology 91, 1163–1171. doi: 10.1890/09-0336.1, PMID: 20462130

[B11] Waqar S. Bhat A. A. Khan A. A. (2024). Endophytic fungi: Unravelling plant-endophyte interaction and the multifaceted role of fungal endophytes in stress amelioration. Plant Physiol. Biochem. 206, 108174. doi: 10.1016/j.plaphy.2023.108174, PMID: 38070242

[B12] Yousuf G. K. (2026). Mitigating drought and heavy metals stress in Triticum aestivum L. using multi-trait tolerant plant growth-promoting endophytic Trichoderma sp. Ghadah-K4. J. Crop Sci. Biotechnol., 1–25. doi: 10.1007/s12892-025-00323-1, PMID: 41822847

